# RBD and Spike DNA-Based Immunization in Rabbits Elicited IgG Avidity Maturation and High Neutralizing Antibody Responses against SARS-CoV-2

**DOI:** 10.3390/v15020555

**Published:** 2023-02-17

**Authors:** Hernan H. M. da Costa, Diego J. B. Orts, Andrew D. Moura, Amaro N. Duarte-Neto, Cinthya S. Cirqueira, Rodrigo A. Réssio, Cristina T. Kanamura, Karen Miguita, Jerenice E. Ferreira, Raimunda T. M. Santos, Patricia P. Adriani, Jair P. Cunha-Junior, Renato M. Astray, Regina M. Catarino, Marcelo Lancelotti, Carlos R. Prudencio

**Affiliations:** 1 Immunology Center, Institute Adolfo Lutz, São Paulo 01246-902, Brazil; 2 Graduate Program Interunits in Biotechnology, University of São Paulo, São Paulo 05508-000, Brazil; 3 Pathology Center, Institute Adolfo Lutz, São Paulo 01246-902, Brazil; 4 Skinzymes Biotechnology Ltd., São Paulo 05441-040, Brazil; 5 Laboratory of Nanopharmaceuticals and Delivery Systems, Department of Pharmacology, Institute of Biomedical Sciences, University of São Paulo, São Paulo 05508-000, Brazil; 6 Laboratory of Immunochemistry and Immunotechnology, Department of Immunology, Federal University of Uberlândia, Uberlândia 38405-317, Brazil; 7 Multi-Purpose Laboratory, Butantan Institute, São Paulo 05503-900, Brazil; 8 Faculty of Pharmaceutical Sciences, Campinas State University, Campinas 13083-871, Brazil

**Keywords:** SARS-CoV-2, COVID-19, DNA immunization, avidity, soroneutralization, antibody response

## Abstract

Neutralizing antibodies (nAbs) are a critical part of coronavirus disease 2019 (COVID-19) research as they are used to gain insight into the immune response to severe acute respiratory syndrome-related coronavirus 2 (SARS-CoV-2) infections. Among the technologies available for generating nAbs, DNA-based immunization methods are an alternative to conventional protocols. In this pilot study, we investigated whether DNA-based immunization by needle injection in rabbits was a viable approach to produce a functional antibody response. We demonstrated that three doses of DNA plasmid carrying the gene encoding the full-length spike protein (S) or the receptor binding domain (RBD) of SARS-CoV-2 induced a time-dependent increase in IgG antibody avidity maturation. Moreover, the IgG antibodies displayed high cross neutralization by live SARS-CoV-2 and pseudoviruses neutralization assays. Thus, we established a simple, low cost and feasible DNA-based immunization protocol in rabbits that elicited high IgG avidity maturation and nAbs production against SARS-CoV-2, highlighting the importance of DNA-based platforms for developing new immunization strategies against SARS-CoV-2 and future emerging epidemics.

## 1. Introduction

The coronavirus disease 2019 (COVID-19) pandemic caused by severe acute respiratory syndrome coronavirus 2 (SARS-CoV-2) has become a serious public health threat worldwide since 2020, when the World Health Organization (WHO) declared a global emergency [1,2,3]. The COVID-19 pandemic imposed new challenges to healthcare services, including requirement for novel immunoassays, immunotherapeutics based on neutralizing antibodies (nAbs), and new vaccinal platforms to assist the public health system and to reduce the increasing number of cases and deaths worldwide [4,5,6,7,8]. Since the onset of the COVID-19 pandemic, several variants of concern (VOCs) have emerged, including Alpha (B.1.1.7), Beta (B.1.351), Gamma (P.1), Delta (B.1.617.2), and the super-spread Omicron (B.1.1.529, BA.1, BA.2, BA.3, BA.4, and BA.5) [9,10]. The major concern about VOCs is related to their ability to increase transmission and/or virulence, and/or to escape current vaccine-induced immunity. Additionally, SARS-CoV-2 Omicron displays significant antigenic drift, and current evidence shows reduction in nAbs titers against Omicrons in subjects who have had prior SARS-CoV-2 infection or in those who received primary immunization [11]. Therefore, the advance of SARS-CoV-2 infections and re-infections requires continuous surveillance studies to characterize important emerging mutations and improve vaccine efficacy [2,8,12,13,14,15,16,17].

Currently, there are many approaches to developing vaccines to protect humans and animals against infectious pathogens. Among the various existing technologies, genetic vaccines (DNA or mRNA) have been formulated with the expectation that they would be flexible, easy to produce, safe and effective [18,19]. mRNA vaccines have shown some challenges, mainly because of RNA inefficiency in in vivo delivery, high instability, and excessive stimulation of inflammatory responses. In addition, the cost of RNA production is high, which may make vaccine development difficult [20].

A DNA-based vaccine platform stands out as a promising candidate for overcoming the bottleneck associated with RNA immunization. DNA vaccination was discovered in the 1990s, when the inoculation of antigen-expressing plasmids induced strong humoral and cellular immune responses [21]. However, to induce these robust immune responses, the gene encoding the antigen must be in the cytoplasmatic environment, where part is processed in the nucleus, which resulting in the presentation of the most relevant peptides on both molecules of major histocompatibility complex classes I and II [22,23]. The great advantage of DNA-based technologies for immunization is the short time required from vaccine design to clinical trials, since these vaccines can be produced at large-scale at low-cost and have high storage stability over a wider temperature range when compared with RNA or viral vector vaccines [21,24,25,26,27]. Thus, it may provide a simple and rapid platform for large-scale production of SARS-CoV-2 nAbs without the need for time-consuming procedures associated with viral isolation and/or production and purification of viral antigens [21,28]. Several DNA and mRNA vaccines expressing mainly the spike protein have been developed against SARS-CoV-2 and are being tested in clinical trials against COVID-19, which have confirmed the strong protective humoral and cellular immune responses in animal models [7,9,24,29,30].

In this pilot study, we establish a DNA-based immunization protocol in rabbits, to evaluate the levels and quality of neutralizing IgG antibodies against SARS-CoV-2. The analysis of immune responses in rabbits demonstrated that our DNA immunization protocol induced high titers of anti-RBD and anti-spike antibodies, and cross protection by neutralization assays. Thus, the DNA-based immunization technology represents a potent tool for the rapid and low-cost production of neutralizing IgG antibodies against SARS-CoV-2, paving the way for the development of new immunoassays and vaccines to combat COVID-19.

## 2. Materials and Methods

### 2.1. Animals and Ethics Statements

Three male New Zealand white rabbits (*Oryctolagus cuniculus*) weighing 2.7 kg were obtained from the Adolfo Lutz Institute (IAL). Rabbits were housed individually at 25 °C and given antibiotic-free food and water *ad libitum*. All protocols were approved in advance by the Adolfo Lutz Institutional Animal Care and Use Committee (CEUA/IAL n^o^. 06-B/2021). All methods were performed in accordance with relevant guidelines and regulations and the ethical principles in animal research adopted by the ARRIVE 2.0 guidelines [31]. We followed the three Rs (3Rs) guiding principles for the more ethical use of animals in scientific research [32].

### 2.2. Production of RBD and Full-Length Spike SARS-CoV-2 Gene Plasmids

The plasmids coding the correspondent gene of the full-length spike protein or the receptor binding domain (RBD) from SARS-CoV-2 were kindly donated by Dr. Florian Krammer, from Icahn School of Medicine, Mount Sinai, New York, NY, U.S.A. [4,33]. Briefly, the sequences used for both proteins were based on the SARS-CoV-2 genomic sequence isolated from Wuhan-Hu-1 (GenBank: MN908947.3) [4]. Sequences were codon-optimized for mammalian cell expression. The full-length spike protein sequence was modified to remove the polybasic cleavage site, which is recognized by furin, and to add a pair of stabilizing mutations. The plasmids were transformed in *E. coli* to be replicated. The freshly transformed colonies were cultured in Luria–Bertani (LB) medium supplemented with ampicillin (100 µg/mL) for 18 h at 37 °C and 250 rpm. High-quality plasmids were obtained using a commercially available PureLink™ Expi Endotoxin-Free Maxi Plasmid Purification Kit (Thermo Fisher Scientific™, Waltham, MA, USA) with an endotoxin-removal step, according to manufacturer’s recommendations.

### 2.3. DNA-Based Immunization by Needle Injection and Serum Samples

For DNA immunization, rabbits were inoculated with three doses by intramuscular route with 0.5 mg, 1 mg, and 0.25 mg of DNA diluted in sterile PBS and 50% (*v*/*v*) of Complete or Incomplete Freund’s Adjuvant for each dose, respectively. In these experiments, plasmids containing either the full-length spike gene insert or the RBD gene insert, and only Freund’s adjuvant as mock control, were used. The plasmid control group was not used due to the limited number of animals available for the experiment. The immunization doses were distributed at 21-day intervals. Blood samples were collected at five time points with the 21-day intervals (day 0–84), and six weeks after the last dose, the rabbits were euthanized. During this procedure, the rabbits were weighed and pre-anesthetic medication using acepromazine 1% at a dosage of 3 mg/kg was injected into the quadriceps muscle. After 5 to 10 min, the anesthetic combination (xylazine at a dose of 7 mg/kg and ketamine at a dose of 35 mg/kg) was injected in the quadriceps muscle. After 15 min, the loss of sensitivity and pain reflexes were verified. Animals were placed in restraint in the prone position and manual trichotomy and asepsis with alcohol were performed for the introduction of the needle (40 × 16) at the thorax close to the xiphoid cartilage. The blood was collected in a sterile bottle for further centrifugation and serum preparation.

### 2.4. Production of RBD and Spike Recombinant Proteins

Plasmids containing RBD and spike gene sequences were transfected in HEK 293F cells (Thermo Fisher Scientific™, Waltham, MA, USA) using a ExpiFectamine 293 Transfection Kit (Thermo Fisher Scientific™, Waltham, MA, USA) to overexpress recombinant proteins. Briefly, 100 mL supernatants from transfected cells were harvested on day 3–5 post-transfection by centrifugation of the culture at 4000× *g* for 20 min, concentrated using an Amicon Ultra Centrifugal Filter (Merck Millipore, Burlington, MA, USA) until the final volume of 5 mL. After, 20 mL of buffer A (sodium phosphate 20 mM, NaCl 500 mM, pH 7.4) was added in the supernatant, and the sample was clarified in a 0.22 µM syringe filter. First, the recombinant proteins were purified by immobilized metal ion affinity chromatography (IMAC) using HisTrap™ Excel columns (GE Healthcare Life Sciences, Chicago, IL, USA) on a fast protein liquid chromatography system (FPLC) (ÄKTA™ Pure, Cytiva Life Sciences, Marlborough, MA, USA). The supernatant was applied to the column, previously equilibrated with buffer A, at a flow rate of 1 mL/min. Unbound proteins were washed away with 10 column volumes (CV) of 20 mM imidazole. Bound fractions were eluted with 20 CV of buffer B (sodium phosphate 20 mM, NaCl 500 mM, Imidazol 500 mM, pH 7.4) using a gradient. The peak corresponding to the protein of interest was collected, concentrated in Amicon centrifugal units (Merck Millipore, Burlington, MA, USA) and re-suspended in 2 mL of phosphate-buffered saline (PBS) for size-exclusion chromatography using a Superdex™ 75 column (Cytiva Life Sciences, Marlborough, MA, USA). The fractions were collected and analyzed by SDS-PAGE 10% to check protein integrity.

### 2.5. Immunoblotting Assay

For Western blot, the 10% SDS-PAGE was transferred overnight to a 0.22 µm nitrocellulose filter membrane (Bio-Rad, Hercules, CA, USA). The membranes were stained with ponceau, divided into four strips, blocked by 5% skim milk (*w*/*v*) diluted in PBS and further incubated with a rabbit anti-His-tag antibody (1:1000, Sigma-Aldrich, St. Louis, MO, USA), the post-third dose rabbit anti-RBD (1:2500) or anti-spike serum (1:2500), or mock group serum (1:2500) diluted in PBS supplemented with 2.5% skim milk (*w*/*v*) for 1 h at room temperature. The strips were washed three times with PBS supplemented with 0.1% Tween 20 (*v*/*v*, PBST 0.1%) followed by incubation with horseradish peroxidase (HRP) labeled goat anti-rabbit IgG (1:8000, Santa Cruz Biotechnology, Dallas, TX, USA) for 1 h at room temperature. After, the strips were washed three times with PBST 0.1% and the membranes were revealed using SuperSignal™ West Pico PLUS Chemiluminescent Substrate (Thermo Fisher Scientific™, Waltham, MA, USA), following the manufacturer’s instructions. Images were recorded using an iBright™ CL1500 Imaging System (Thermo Fisher Scientific™, Waltham, MA, USA).

### 2.6. Pathological Examination and Histochemical Staining

Lung and spleen samples were collected from the necropsies of rabbits after immunization. All specimens were fixed with 4% neutral buffered formaldehyde, processed with alcohol and xylene, embedded in paraffin wax, and 3 µm sections were cut. Sections were stained with hematoxylin and eosin (HE) according to the standard protocol. Histological analysis of the immunized and control rabbits was performed using an optical microscope.

### 2.7. Indirect ELISA for Anti-RBD and Anti-Spike Specific IgG Antibodies Detection and Avidity Maturation Evaluation

Rabbit anti-RBD and anti-spike IgG antibodies were detected by enzyme immunoassay (ELISA) using an adapted protocol previously described by our group [8]. To ensure test reproducibility and concordance, two ELISA using the same protocol were performed on two different days. The average of all results from both days was used for plotting the graphs. Briefly, high-binding 96-well plates (Nunc MaxiSorp™ flat-bottom, Thermo Fisher Scientific™, Waltham, MA, USA) were coated with 50 µL per well of 2.5 µg/mL of recombinant RBD or spike protein diluted in PBS and incubated at 4 °C overnight. After, plates were washed six times with PBS supplemented with 0.5% Tween 20 (PBST 0.5%). All washing steps were performed using an ELISA plate washer (Washwell plate, Robonik, Thane, India). After, 200 µL per well of 5% skim milk powder diluted in PBST 0.5% as a blocking solution was added to the plates and incubated for 2 h at 37 °C. After blocking, plates were washed six times with PBST 0.5% and incubated with 100 µL per well of rabbit-immunized serum samples, in duplicate, diluted 1:50 or 1:100, for RBD and spike, respectively, in PBST 0.5% supplemented with 1% skim milk for 1 h at 37 °C. Next, plates were washed six times with PBST 0.5% and then half of the plate was incubated in the presence of potassium thiocyanate (KSCN) 1.5 M and half of the plate in the presence of PBST 0.5% (100 µL/well) for 20 min at 37 °C. Plates were washed six times and incubated with 50 µL of a 1:5000 dilution of goat anti-rabbit IgG−horseradish peroxidase (HRP) conjugated antibody (Santa Cruz Biotechnology, Dallas, TX, USA) diluted in PBST 0.5% supplemented with 1% skim milk for 1 h at 37 °C. Plates were washed again and incubated for 10 min with 100 µL/well of One Step-TMB (3,3′,5,5′-tetramethylbenzidine) (Scienco, Santa Catarina, Brazil). The reaction was stopped by the addition of 50 µL per well of 1 N sulfuric acid. The absorbance at 450 nm, using 630 nm as a reference, was measured using a Multiskan MS plate reader (Labsystems). The pre-immune serum absorbances were used to calculate the cut-off, using the mean plus three standard deviation. The avidity index (AI) was expressed as follows: absorbance mean value from KSCN treated sample divided by the absorbance mean value from the nontreated samples and multiplied by 100%.

### 2.8. Neutralization Assay against Live SARS-CoV-2 Viruses

SARS-CoV-2 isolate was provided by the Adolfo Lutz Institute to determine the neutralizing titer and propagate in Vero cells [34]. Briefly, twofold serially diluted serum solutions (from 1:20 to 1:80) were incubated with 1000 PFU of SARS-CoV-2 B8 strain for one hour at 37 °C. Antibody-virus complexes were added to Vero cell monolayers in 24-well plates and incubated for 24 h. The positive control was Vero cells’ infection with 1000 PFU of SARS-CoV-2 B8 in the absence of serum. After incubation, the total RNA of the monolayer containing Vero cells and SARS-CoV-2 was extracted by PureLink™ RNA Mini Kit (Ambion^®^ Carlsbad, CA, USA). The neutralizing activity of the antibodies against SARS-CoV-2 was determined by qRT–PCR using a standard curve of the amplicon ESobecco primers [35] in a SYBRGreen detection system (Promega, Madison, WI, USA). The copy number was determined as described previously [34].

### 2.9. SARS-CoV-2 Pseudotyped Virus Neutralization Assays

The plasmids were kindly provided by Dr. Gary R. Whittaker from the Department of Microbiology and Immunology, College of Veterinary Medicine, Cornell University. The system contained the following plasmids: pCMV-MLVgagpol plasmid (MLV gag and pol-encoding), pTG-Luc transfer vector with luciferase reporter, pCAGGS-VSVG plasmid, pCAGGS plasmid (deltaEnv) and pcDNA-SARS-CoV-2-S [36]. Briefly, pseudoviruses carrying the Wuhan variant spike glycoprotein and a firefly luciferase (FLuc) reporter gene were produced in HEK 293 T cells. Pseudovirus supernatants were collected 48 h after transfection, filtered through a 0.45 µm filter and used immediately. Pseudovirus neutralization titers were measured by infecting Vero cells for 48 h. Briefly, pseudoviruses with titers of approximately 10^4^ of Relative Luminescence Units per milliliter of pseudovirus supernatant (RLU/mL) of luciferase activity were incubated with anti-RBD and anti-spike serum for 1 h at 37 °C. Mixtures of pseudoviruses and two-fold serially diluted serum solutions (from 1:10 to 1:40), in duplicate, were then inoculated into 96-well plates containing 3 × 10^4^ Vero cells per well. Pseudovirus infectivity was assessed after 48 h using the Luciferase Assay System kit (Promega, Madison, WI, USA). Pseudovirus neutralization titers were expressed as RLU/mL.

### 2.10. Statistical and Data Analysis

Statistical analyses and graphics were performed using the GraphPad Prism v. 8.0.1 (GraphPad Software, San Diego, CA, USA). The values of RNA copy number from SARS-CoV-2 in the neutralizing test were evaluated for normal distribution by Shapiro–Wilk normality test. The antibody dilutions were compared with the control and evaluated by Brown–Forsythe and Welch ANOVA test with Dunnett’s multiple comparation. *p* values < 0.05 were considered statistically significant.

## 3. Results

### 3.1. Plasmid Purification and Antigen Production

**Figure 1:**
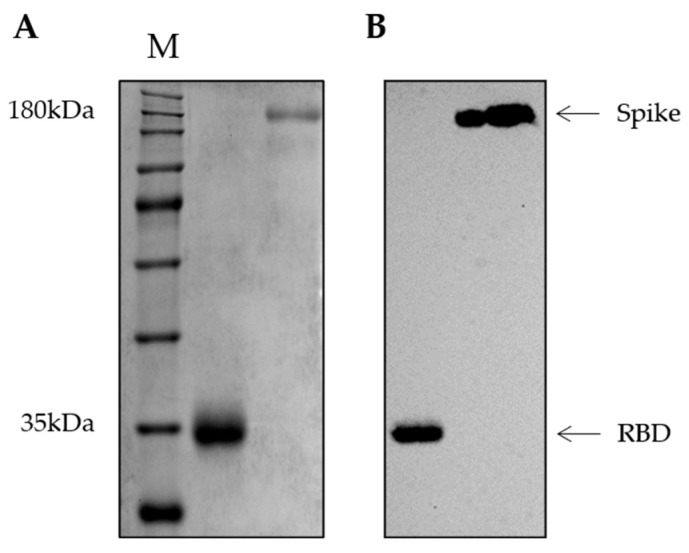
RBD and full-length spike recombinant proteins purification. (**A**) 10% SDS-PAGE in denaturing conditions showing the RBD and full-length spike purification. (**B**) Western blot of RBD and spike purified fractions, using commercial rabbit anti-His-tag antibody (1:1000, M: TrueColor High Range Protein Marker); The images from SDS-PAGE and blot were adjusted for brightness and contrast and were recorded.

The SARS-CoV-2 DNA constructs were generated by introducing human codon-optimized sequences encoding the full-length spike (S) and receptor binding domain (RBD) proteins from the Wuhan strain into a pCAGGS mammalian expression vector. Plasmids were amplified in *E. coli* and purified. Additionally, using the same plasmids, we expressed RBD and spike proteins in human embryonic kidney (HEK) Expi293F cells and purified the protein using a two-step protocol. Purified soluble proteins were resolved by 10% sodium dodecyl sulfate-polyacrylamide gel electrophoresis (SDS-PAGE) under denaturing conditions that showed a ~32 kDa protein corresponding to RBD and a 190 kDa corresponding to full spike (Figure 1A). Western blot using a commercial rabbit anti-His-tag antibody at 1:1000 confirmed the presence of recombinant proteins at approximately 32 kDa and 190 kDa for RBD and spike, respectively (Figure 1B).

### 3.2. Vaccination with a DNA Plasmid by Needle Injection Elicits Robust Lymphoid Proliferation in the Spleen and Lung-Associated Tissues and High Titers of Anti-RBD and Anti-Spike IgG in Rabbits

**Figure 2:**
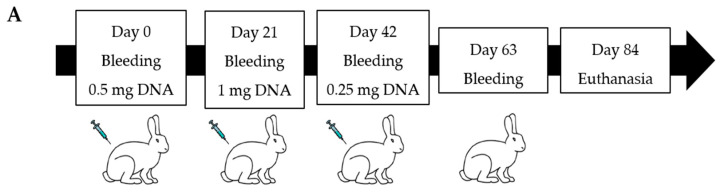

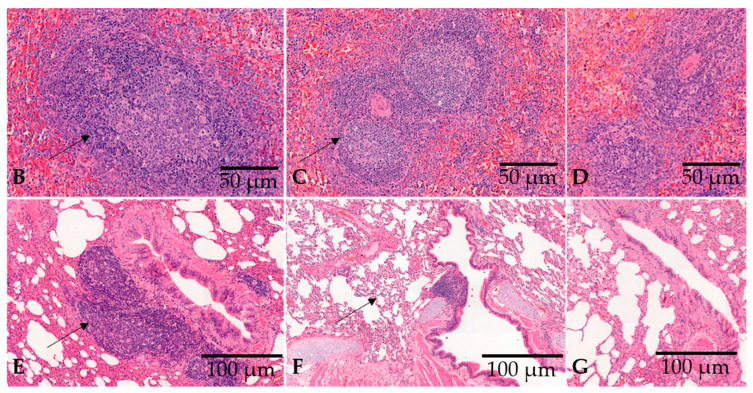
Rabbit RBD and spike DNA-based immunization. (**A**) Rabbit immunization schedule. (**B**–**G**) Histopathology of spleen and lungs. The immunized rabbits showed lymphoid hyperplasia in the spleen white pulp and in the bronchus-associated lymphoid tissue (BALT) (black arrows). (**B**,**E**): spleen and lung from RBD immunized rabbit, respectively. (**C**,**F**): spleen and lung from spike immunized rabbit, respectively. (**D**,**G**): spleen and lung from control animal, respectively. Mock animal did not show germinative centers in the spleen white pulp and the BALT was minimal in the lungs. Scale bars: (**B**–**D**) = 50 µm, (**E**–**G**) = 100 µm.

Three DNA plasmid doses (0.5 mg, 1 mg, 0.25 mg) and five bleeding points were established accordingly to an immunization schedule (Figure 2A). After the animals’ euthanasia, a histopathological analysis was performed on the spleen and lung tissue sections, stained with hematoxylin and eosin (HE). Immunized animals showed pronounced lymphoid tissues hyperplasia in the spleen white pulp (Figure 2B–D) and in the bronchus-associated lymphoid tissue (BALT) in the lungs (Figure 2E–G). The mock animal showed lymphoid tissue without germinative centers. Animals did not show acute lung injury or eosinophilic inflammatory infiltrates in organs after the DNA-based immunization protocol.

### 3.3. Vaccination with a DNA Plasmid Elicits High Titers of Anti-RBD and Anti-Spike IgG in Rabbits

**Figure 3:**
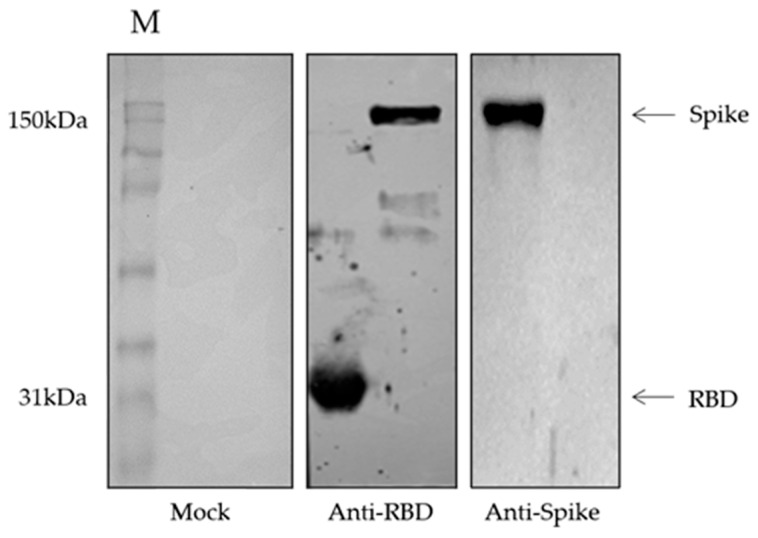
Western blot of recombinant RBD and spike purified fractions using post-third dose rabbit anti-RBD or anti-spike antibody (1:2500) to assess immunoreactivity to the recombinant proteins. The mock group received only the Freund’s adjuvants in the immunization. M: Rainbow Marker—Full range; blot images were adjusted for brightness and contrast and were recorded.

To assess the immunoreactivity of animal’s sera after the immunization schedule, we first performed a Western blot using the post-third dose of anti-RBD and anti-spike rabbit serum and purified RBD and spike proteins. Anti-RBD serum showed reactivity against both RBD and spike, whereas the anti-spike serum showed the reactivity only against the spike protein. The mock animal immunized with Freund’s adjuvants showed no reactivity against both RBD and spike proteins (Figure 3).

**Figure 4:**
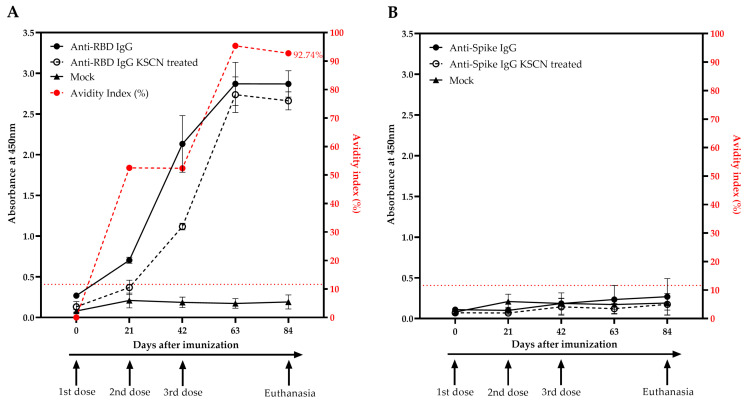

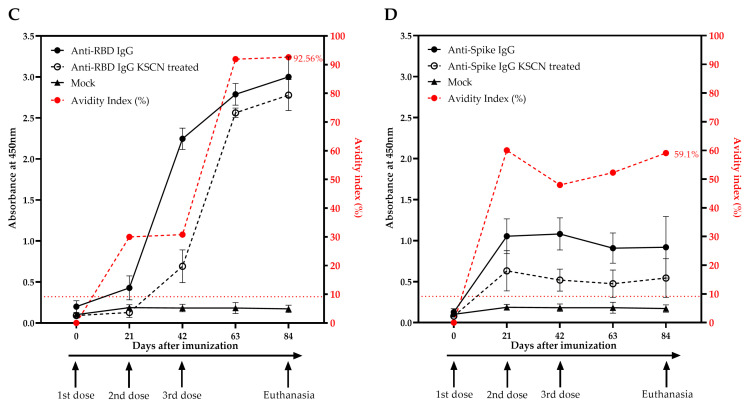
RBD- and spike-specific IgG antibody levels and avidity index against SARS-CoV-2. Evaluation of: (**A**) anti-RBD and (**B**) anti-spike specific-IgG and avidity against RBD as a coating protein; and (**C**) anti-RBD and (**D**) anti-spike specific-IgG and avidity against spike as coating protein. Levels of anti-RBD and anti-spike IgG (continuous black lines and dots), anti-RBD and anti-spike IgG after KSCN treatment (discontinuous black lines and circles), and mock group IgG (continuous black line and triangle). The avidity index is shown in discontinuous red lines and dots. The ELISA data are expressed as the absorbance at 450 nm and the dotted red line shows the cut-off value.

Moreover, an in-house ELISA using RBD and spike proteins as coat antigens was performed to detect RBD- and/or spike-specific IgG in serum post-immunization. The mock group immunized serum showed no reactivity against RBD and spike proteins. When using the RBD as a coat antigen, a positive IgG reaction was observed in the rabbit immunized with DNA encoding RBD just after the first dose. The immunoreactivity of anti-RBD IgG increased over the weeks after the second dose, reaching the highest reactivity 21 days after the third dose and maintaining this level for 42 days after the last dose (Figure 4A). In contrast, the animal immunized with the DNA encoding the full-length spike protein showed no reactivity against the RBD protein as coat antigen after any dose (Figure 4B).

The anti-RBD and anti-spike IgG antibodies were also evaluated for the binding strength of antibodies to the RBD protein as a coat antigen, measured as avidity index (AI). The rabbit immunized with DNA encoding RBD showed increasing levels of avidity 21 days after the first dose, reaching 52.5% at this point and continuing to increase over the next weeks, reaching AI = 92.74% in the last week (Figure 4A). When anti-spike IgG was evaluated in ELISA using the RBD as coat antigen, no avidity measurement was detected, since serum from animals immunized with spike did not display reactivity to the RBD molecule (Figure 4B). Interestingly, when the avidity assay was performed with anti-RBD serum using the spike protein as a coat antigen, a similar RBD avidity profile was obtained (AI = 92.56% against spike) (Figure 4C). However, serum from rabbit immunized with DNA encoding the full-length spike showed reactivity to spike protein after the first dose and maintaining the antibody levels after further doses. The avidity of anti-spike serum reached 59.1% on the day of euthanasia (Figure 4D). The ELISA results were in concordance with Western blotting analysis using the same serum sample.

### 3.4. Neutralization Potential of Anti-RBD and Anti-Spike IgG Antibodies against Live and Pseudotyped SARS-CoV-2 Particles

**Figure 5:**
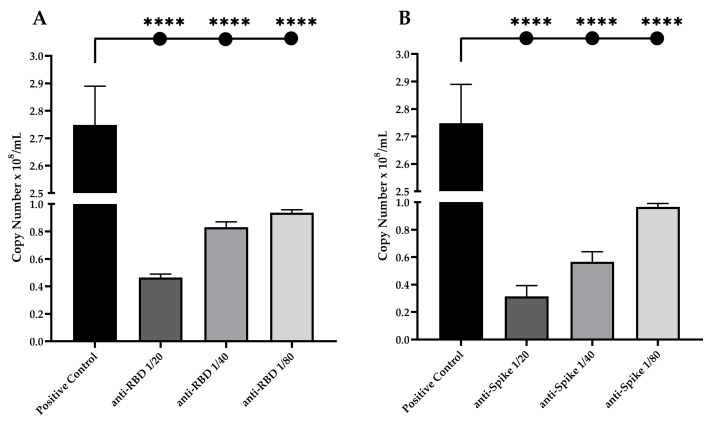
Serum neutralization expressed in copy number of SARS-CoV-2 B8 strain particles detected in a total amount of 1 µg of RNA. Twofold serially diluted (from 1:20 to 1:80) solutions of (**A**) anti-RBD or (**B**) anti-spike antibodies were incubated with 1000 PFU of SARS-CoV-2 B8 strain for one hour at 37 °C. Asterisks represent significant comparisons between positive control and serum treated cells (**** *p* < 0.0001).

To evaluate the neutralization potential of anti-RBD and anti-spike IgG antibodies, both pseudovirus assay and live viral neutralization assay were performed. The SARS-CoV-2 B8 strain was used to evaluate the presence of neutralizing IgG antibodies. Serial dilutions of anti-RBD rabbit serum (1:20; 1:40 and 1:80) showed a significant reduction in live virus infection, of up to ~83% inhibition at 1:20 dilution (*p* < 0.0001), ~70% inhibition at 1:40 dilution (*p* < 0.0001), and ~66% inhibition at 1:80 dilution (*p* < 0.0001), when compared with the positive control (Figure 5A). When using the anti-spike rabbit serum, we observed a slightly higher reduction in virus infection, up to ~88.5% inhibition at 1:20 dilution (*p* < 0.0001), ~79% inhibition at 1:40 dilution (*p* < 0.0001), and ~65% inhibition at 1:80 dilution (*p* < 0.0001), when compared with the positive control (Figure 5B).

**Figure 6:**
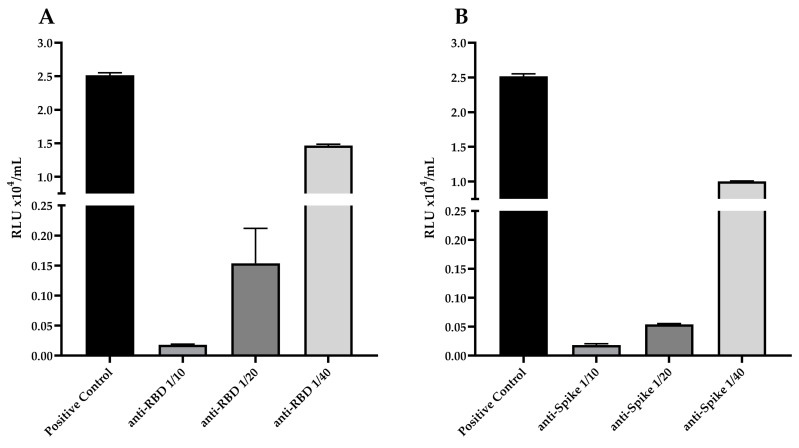
Serum neutralization of SARS-CoV-2 pseudoviruses particles. Two-fold serially diluted (from 1:10 to 1:40) solutions of (**A**) anti-RBD or (**B**) anti-spike serum were incubated with pseudoviruses with titers of approximately 10^4^ RLU/mL of luciferase activity for one hour at 37 °C. Pseudovirus infectivity was assessed using the Luciferase Assay. Pseudovirus neutralization titers are expressed as RLU/mL.

When using pseudotyped SARS-CoV-2 particles, serial dilutions of anti-RBD rabbit serum (1:10; 1:20 and 1:40) showed a significant reduction in virus infection, up to ~99% inhibition at 1:10 dilution, ~93% inhibition at 1:20 dilution, and ~42.5% inhibition at 1:40 dilution, when compared with the positive control (Figure 6A). When using the anti-spike rabbit serum, we observed a reduction in virus infection of up to ~99% inhibition at 1:10 dilution, ~97% inhibition at 1:20 dilution, and ~58% inhibition at 1:40 dilution, when compared with the positive control (Figure 6B).

## 4. Discussion

SARS-CoV-2 exhibits high infectivity capacity and has reached millions of cases worldwide, with millions of deaths observed [16,37,38]. Brazil has faced a rapid escalation of COVID-19 cases and almost seven hundred thousand registered deaths. Despite the reduction in the number of cases and deaths in Brazil, new waves could emerge at any time, considering the emergence of new SARS-CoV-2 super spreading variants that may escape from the immune response or the available vaccines or drugs [10,37,38,39,40]. Several studies have reported the antibody response profile to SARS-CoV-2, which includes the broad clinical and vaccinology spectrum of COVID-19, however, many aspects of the humoral immune response in COVID-19 remain obscure in both situations [17,21,29,37,41].

Vaccines tailored using integrated approaches are urgently needed to address existing and new challenges in the combat against human infectious diseases. Multiple vaccine candidates are still under development and approval by regulatory agencies. In an attempt to understand the potential use of the DNA-based strategy to produce high quality neutralizing antibodies, we proposed a pilot immunization scheme by needle injection of rabbits based on plasmids encoding the full-length spike or the receptor binding domain (RBD) genes. This type of immunization allowed the generation of high-quality neutralizing IgG antibodies and provided several advantages over other approaches, such as their biosafety because there is no infection by pathogens, since attenuated or inactivated SARS-CoV-2 viruses are not necessary. In addition, the antigens maintain their native conformation since not undergoing in vitro protein production and purification [21,28]. Additionally, the versatility of DNA vaccines as a multivalent vaccine development platform is a plus that makes them excellent to test in vaccine candidates for several diseases, mainly for ongoing emerging infectious diseases [24,25,26].

Naked plasmid DNA is the most straightforward, consisting of circular double-stranded DNA that is simple to manipulate and can easily be produced in large quantities from bacterial culture [21]. Additionally, rabbits are a useful model to study the immunogenicity of DNA-based vaccines, particularly for the study of antibody responses once they display a strong immune response to various vaccines producing high affinity antibodies [21,42,43,44]. The histopathology of the immunized rabbit organs showed lymphoid hyperplasia with formation of germinative centers in the spleen, indicating a strong immune cellular activation (Figure 2B–G). Germinal centers where mature B cells are activated are responsible for affinity maturation and consist of antigen-specific B cells that undergo proliferation, somatic hypermutation and subsequent selection [45].

Additionally, our results showed that DNA-based immunization elicited a similar immunoreactivity of anti-RBD profile both using RBD and spike as antigen in ELISA (Figure 4A,C). In contrast, the animal immunized with DNA encoding the full-length spike gene showed no reactivity against the RBD coating protein after any dose, but showed reactivity against the spike protein after the first dose and maintained the antibody levels after the next doses (Figure 4B,D). The Western blot results using the post-immunization rabbit serum corroborated the ELISA results, in which only the RBD-DNA immunization raised an immune response to both proteins, whereas the spike-DNA immunization only elicited an immune response to the spike full protein (Figure 3).

The spike protein is divided into two subunits, S1 and S2. The RBD on the S1 subunit displays an essential role in binding to the receptor on the surface of host cell. Previous works have demonstrated that RBD protein is immunodominant in rabbits when compared with other spike immunogen domains (S1, S2, or S1 + S2), eliciting a higher titer of high affinity antibodies. However, there was no detection of RBD in the Western blot assay, suggesting that RBD may form a complicated conformation under both native and denaturing conditions, as previous reports [46,47]. Allosteric propensities of SARS-CoV-2 allow for the efficient modulation of complex phenotypic responses to the host receptor and antibodies [48].

Moreover, the lack of RBD recognition by the rabbit anti-spike serum might be related to a specific antigenic presentation effect of the full-length spike in its complete form allowing the hidden RBD region to not be properly recognized by the immune system. It has been described that RBD protein suffers spontaneous structural fluctuation between an ‘up’ and a ‘down’ conformation, but only the up conformation enables the exposure of the RBD binding motif (RBM), which can become accessible to bind in the host receptor ACE2 or for recognition by the immune system [15,37,49]. Changes can be undertaken by the design of the complete full-length spike gene in order to improve the RBD antigenic presentation, and also favor the accessibility of RBD epitopes to increase the specificity of B cell immune responses or binding to the ACE2 receptor [50,51,52,53].

Additionally, the rabbit anti-spike serum was able to generate a strong neutralizing effect, similar to the effect generated by immunization with the RBD gene which also suggested that immune response was focused to a region other than RBD, such as the N-terminal domain (NTD). The NTD has been described as an important region for neutralization of SARS-CoV-2 or other CoVs, where antibodies may cause conformational changes in the spike protein, that are required for fusion or interactions with cell receptors, in both live virus and pseudovirus assays [54,55,56,57]. If this proves to be true, a focus away from the RBD may be highly desirable for protection against multiple VOCs. However, further studies are needed, such as epitope mapping or heterologous immunization, to address these questions [21,29,58,59].

Furthermore, our vaccination protocol showed a time and dose-dependent increase in the avidity antibody maturation (Figure 4) and, interestingly, there was a possible association between SARS-CoV-2 antibody avidity with the high nAbs titers for RBD and spike (Figure 4, Figure 5 and Figure 6). Avidity indices below 40% are considered as low levels, between 40% and 55% is considered to represent an intermediate phase or “maturation zone”, and above 55% is considered high avidity, in which the correlation between avidity and soroneutralization activity suggests protection [30,41,60]. Most cases of SARS-CoV-2 natural infections usually lead to incomplete avidity maturation, characterized by low or intermediate avidity. In contrast, it was reported that two vaccination steps with BioNTech mRNA vaccine allowed complete avidity maturation, leading to high avidity in most cases [30,61]. High-avidity antibodies are capable of blocking SARS-CoV-2 receptor binding, protecting and promoting virus neutralization, which indicates that avidity antibodies maturation might be associated with the production of protective nAbs in viral infections [41,60,62,63].

Our previous study comparing mild and severe symptomatic patients concluded that severe patients had higher levels of anti-RBD IgG antibodies than mild patients. Moreover, IgG antibody avidity was low (AI up to 30%) during the initial infection and increased during time after the onset of the illness, mainly in the severe patients who survived the disease compared with those who died, which may lead to the hypothesis that a severe COVID-19 illness may enhance avidity maturation, but the failure to reach higher avidity levels increases the risk of death [8,61]. Therefore, it is extremely necessary that novel vaccines can elicit a long-lasting immunity activity with high avidity levels and nAbs titers, and, thus, elicit a stronger immune response than natural infections [30,60,64]. Thus, the monitoring of IgG avidity may contribute to vaccination success since it is well established that affinity/avidity maturation depends on the persistence and availability of the antigen throughout the maturation process [30,65]. Efficient neutralization of SARS-CoV-2 would require antibodies of high avidity, adding to our classic concept of neutralizing antibodies, the additional requirement for high affinity/avidity [30,66,67], as new SARS-CoV-2 VOC seem to bind to ACE2 with higher affinity than the original isolate of SARS-CoV-2 [30,39,68,69,70].

In the end, the serum obtained from vaccinated rabbit was able to neutralize SARS-CoV-2 in vitro, determined similarly by both soroneutralization and pseudovirus assays. However, live SARS-CoV-2 neutralization assays demonstrated that rabbit serum was able to reduce about three times the quantity of SARS-CoV-2 genome production in Vero cells in comparison with our previous work [34], in which vaccinated OMV/ZIKV mice serum reduced about six times the Zika virus infection in Vero cells. In addition, in the pseudovirus assay, the titers of 10^4^ RLU/mL of luciferase activity in Vero cells were comparable to those described by Millet et al. (2019) [36], which exhibited consistent data about the pseudovirus infectivity, in which the inhibition of luciferase entry in cells were similar to the live virus inhibition. The success of the antibodies production and the soroneutralization indicated an efficient RBD and spike DNA-based vaccine for virus described in this work.

One of the limitations of this study was the use of a small number of animals in the immunizations because of the scope of this initial research project. This study will be a starting point for other studies in the future as we successfully demonstrate the feasibility of DNA-based immunizations. Based on this, we hope to undertake a continuity of this study covering a larger number of animals and testing different immunization conditions, and improve soroneutralization assays, including the new VOCs, in the improvement of formulations for more efficient delivery of plasmids in the cell precisely with the modulation of the desirable immune response for protection.

## 5. Conclusions

In summary, the results reported here demonstrated that DNA-based immunization in rabbits elicited high nAb responses and increased IgG avidity maturation over time against SARS-CoV-2. These results addressed an important question in COVID-19 vaccine research by offering a simple, cheap and effective method to produce neutralizing IgG antibodies specific to SARS-CoV-2. Additionally, it provided a novel and simple approach for monitoring the high IgG affinity/avidity, which may contribute to the success of immunization. Additionally, this approach will provide the production of polyclonal antibodies as diagnostic reagents for immunoassays. Moreover, we might conclude that DNA-based immunization in rabbits is a viable approach to produce functional antibody responses and highlights this platform for developing new vaccine strategies against SARS-CoV-2 and variants of concern (VOCs) in the near future.

## Data Availability

Not applicable.

## References

[1] Zheng J. (2020). SARS-CoV-2: An Emerging Coronavirus That Causes a Global Threat. Int. J. Biol. Sci..

[2] Zhang J.J., Dong X., Cao Y.Y., Yuan Y.D., Yang Y.B., Yan Y.Q., Akdis C.A., Gao Y.D. (2020). Clinical Characteristics of 140 Patients Infected with SARS-CoV-2 in Wuhan, China. Allergy Eur. J. Allergy Clin. Immunol..

[3] Chen N., Zhou M., Dong X., Qu J., Gong F., Han Y., Qiu Y., Wang J., Liu Y., Wei Y. (2020). Epidemiological and Clinical Characteristics of 99 Cases of 2019 Novel Coronavirus Pneumonia in Wuhan, China: A Descriptive Study. Lancet.

[4] Stadlbauer D., Amanat F., Chromikova V., Jiang K., Strohmeier S., Arunkumar G.A., Tan J., Bhavsar D., Capuano C., Kirkpatrick E. (2020). SARS-CoV-2 Seroconversion in Humans: A Detailed Protocol for a Serological Assay, Antigen Production, and Test Setup. Curr. Protoc. Microbiol..

[5] Gaspar E.B., Prudencio C.R., De Gaspari E. (2021). Experimental Studies Using OMV in a New Platform of SARS-CoV-2 Vaccines. Hum. Vaccines Immunother..

[6] Jiang S., Hillyer C., Du L. (2020). Neutralizing Antibodies against SARS-CoV-2 and Other Human Coronaviruses. Trends Immunol..

[7] Li Y., Bi Y., Xiao H., Yao Y., Liu X., Hu Z., Duan J., Yang Y., Li Z., Li Y. (2021). A Novel DNA and Protein Combination COVID-19 Vaccine Formulation Provides Full Protection against SARS-CoV-2 in Rhesus Macaques. Emerg. Microbes Infect..

[8] Moura A.D., da Costa H.H.M., Correa V.A., Ana A.K., Lindoso J.A.L., De Gaspari E., Hong M.A., Cunha-Junior J.P., Prudencio C.R. (2021). Assessment of Avidity Related to IgG Subclasses in SARS-CoV-2 Brazilian Infected Patients. Sci. Rep..

[9] Li J., Liu Q., Liu J., Fang Z., Luo L., Li S., Lei Y., Li Z., Jin J., Xie R. (2022). Development of Bivalent MRNA Vaccines against SARS-CoV-2 Variants. Vaccines.

[10] Takashita E., Yamayoshi S., Simon V., van Bakel H., Sordillo E.M., Pekosz A., Fukushi S., Suzuki T., Maeda K., Halfmann P. (2022). Efficacy of Antibodies and Antiviral Drugs against Omicron BA.2.12.1, BA.4, and BA.5 Subvariants. N. Engl. J. Med..

[11] WHO Enhancing Response to Omicron SARS-CoV-2 Variant: Technical Brief and Priority Actions for Member States. https://www.who.int/publications/m/item/enhancing-readiness-for-omicron-(b.1.1.529)-technical-brief-and-priority-actions-for-member-states.

[12] Santos F.R.D.S., de Azevedo M.S.P., Bielavsky M., da Costa H.H.M., Ribeiro D.G., Nascimento G.G.D., Marcondes G.M.P., de Castro B.P., de Lima Neto D.F., Prudencio C.R. (2021). Mutational Profile Confers Increased Stability of SARS-CoV-2 Spike Protein in Brazilian Isolates. J. Biomol. Struct. Dyn..

[13] Astuti I. (2020). Ysrafil Severe Acute Respiratory Syndrome Coronavirus 2 (SARS-CoV-2): An Overview of Viral Structure and Host Response. Diabetes Metab. Syndr. Clin. Res. Rev..

[14] Tai W., He L., Zhang X., Pu J., Voronin D., Jiang S., Zhou Y., Du L. (2020). Characterization of the Receptor-Binding Domain (RBD) of 2019 Novel Coronavirus: Implication for Development of RBD Protein as a Viral Attachment Inhibitor and Vaccine. Cell. Mol. Immunol..

[15] Walls A.C., Park Y.J., Tortorici M.A., Wall A., McGuire A.T., Veesler D. (2020). Structure, Function, and Antigenicity of the SARS-CoV-2 Spike Glycoprotein. Cell.

[16] Wei Y., Lu Y., Xia L., Yuan X., Li G., Li X., Liu L., Liu W., Zhou P., Wang C.Y. (2020). Analysis of 2019 Novel Coronavirus Infection and Clinical Characteristics of Outpatients: An Epidemiological Study from a Fever Clinic in Wuhan, China. J. Med. Virol..

[17] Yang H.S., Racine-Brzostek S.E., Lee W.T., Hunt D., Yee J., Chen Z., Kubiak J., Cantu M., Hatem L., Zhong E. (2020). SARS-CoV-2 Antibody Characterization in Emergency Department, Hospitalized and Convalescent Patients by Two Semi-Quantitative Immunoassays. Clin. Chim. Acta.

[18] Porter K.R., Raviprakash K. (2017). DNA Vaccine Delivery and Improved Immunogenicity. Curr. Issues Mol. Biol..

[19] Pardi N., Hogan M.J., Weissman D. (2020). Recent Advances in MRNA Vaccine Technology. Curr. Opin. Immunol..

[20] Pardi N., Muramatsu H., Weissman D., Karikó K. (2013). In Vitro Transcription of Long RNA Containing. Methods Mol. Biol..

[21] Wang S., Lu S. (2013). DNA Immunization. Curr. Protoc. Microbiol..

[22] Dey A., Rajanathan T.M.C., Chandra H., Pericherla H.P.R., Kumar S., Choonia H.S., Bajpai M., Singh A.K., Sinha A., Saini G. (2021). Immunogenic Potential of DNA Vaccine Candidate, ZyCoV-D against SARS-CoV-2 in Animal Models. Vaccine.

[23] Liu M.A. (2003). DNA Vaccines: A Review. J. Intern. Med..

[24] Excler J.L., Saville M., Berkley S., Kim J.H. (2021). Vaccine Development for Emerging Infectious Diseases. Nat. Med..

[25] Jiang J., Ramos S.J., Bangalore P., Elwood D., Cashman K.A., Kudchodkar S.B., Schultheis K., Pugh H., Walters J., Tur J. (2021). Multivalent DNA Vaccines as a Strategy to Combat Multiple Concurrent Epidemics: Mosquito-Borne and Hemorrhagic Fever Viruses. Viruses.

[26] Matić Z., Šantak M. (2022). Current View on Novel Vaccine Technologies to Combat Human Infectious Diseases.

[27] Maslow J.N. (2018). The Cost and Challenge of Vaccine Development for Emerging and Emergent Infectious Diseases. Lancet.

[28] WHO W.H.O. (2007). WHO Expert Committee on Biological Standardization.

[29] Silveira M.M., Moreira G.M.S.G., Mendonça M. (2021). DNA Vaccines against COVID-19: Perspectives and Challenges. Life Sci..

[30] Bauer G. (2022). High Avidity of Vaccine-Induced Immunoglobulin G against SARS-CoV-2: Potential Relevance for Protective Humoral Immunity. Explor. Immunol..

[31] du Sert N.P., Hurst V., Ahluwalia A., Alam S., Avey M.T., Baker M., Browne W.J., Clark A., Cuthill I.C., Dirnagl U. (2020). The Arrive Guidelines 2.0: Updated Guidelines for Reporting Animal Research. PLoS Biol..

[32] Russell W.M.S., Burch R.L. (1959). The Principles of Humane Experimental Technique.

[33] Amanat F., Stadlbauer D., Strohmeier S., Nguyen T.H.O., Chromikova V., McMahon M., Jiang K., Arunkumar G.A., Jurczyszak D., Polanco J. (2020). A Serological Assay to Detect SARS-CoV-2 Seroconversion in Humans. Nat. Med..

[34] Martins P., Machado D., Theizen T.H., Guarnieri J.P.O., Bernardes B.G., Gomide G.P., Corat M.A.F., Abbehausen C., Módena J.L.P., Melo C.F.O.R. (2018). Outer Membrane Vesicles from Neisseria Meningitidis (Proteossome) Used for Nanostructured Zika Virus Vaccine Production. Sci. Rep..

[35] Corman V., Landt O., Kaiser M., Molenkamp R., Meijer A., Chu D.K., Bleicker T., Brünink S., Schneider J., Luisa Schmidt M. (2020). Detection of 2019 -NCoV by RT-PCR. Euro Surveill.

[36] Millet J.K., Tang T., Nathan L., Jaimes J.A., Hsu H.-L., Daniel S., Whittaker G.R. (2019). Production of Pseudotyped Particles to Study Highly Pathogenic Coronaviruses in a Biosafety Level 2 Setting. J. Vis. Exp..

[37] Qi H., Liu B., Wang X., Zhang L. (2022). The Humoral Response and Antibodies against SARS-CoV-2 Infection. Nat. Immunol..

[38] Andreano E., Paciello I., Pierleoni G., Maccari G., Antonelli G., Abbiento V., Pileri P., Benincasa L., Giglioli G., Piccini G. (2022). MRNA Vaccines and Hybrid Immunity Use Different B Cell Germlines to Neutralize Omicron BA.4 and BA.5. bioRxiv.

[39] He X., Hong W., Pan X., Lu G., Wei X. (2021). SARS-CoV-2 Omicron Variant: Characteristics and Prevention. MedComm.

[40] Wang Q., Guo Y., Iketani S., Nair M.S., Li Z., Mohri H., Wang M., Yu J., Bowen A.D., Chang J.Y. (2022). Antibody Evasion by SARS-CoV-2 Omicron Subvariants BA.2.12.1, BA.4 and BA.5. Nature.

[41] Chan P.K.S., Lim P.L., Liu E.Y.M., Cheung J.L.K., Leung D.T.M., Sung J.J.Y. (2005). Antibody Avidity Maturation during Severe Acute Respiratory Syndrome-Associated Coronavirus Infection. J. Infect. Dis..

[42] Zhang T., Zhang J., Cui X., Zheng J., Li R., Wang F., Liu J., Hu Y.-H. (2017). Evaluation of Immune Protection Induced by DNA Vaccines from Haemaphysalis Longicornis Paramyosin in Rabbits. Parasit. Vectors.

[43] Burgain A., Rochard A., Trollet C., Mazuet C., Popoff M.R., Escriou V., Scherman D., Bigey P. (2013). DNA Electroporation in Rabbits as a Method for Generation of High-Titer Neutralizing Antisera Examples of the Botulinum Toxins Types A, B, and E. Hum. Vaccines Immunother..

[44] Chen Y., Zhu L., Huang W., Tong X., Wu H., Tao Y., Tong B., Huang H., Chen J., Zhao X. (2021). Potent RBD-Specific Neutralizing Rabbit Monoclonal Antibodies Recognize Emerging SARS- CoV-2 Variants Elicited by DNA Prime-Protein Boost Vaccination Emerging SARS-CoV-2 Variants Elicited by DNA Prime-Protein Boost. Emerg. Microbes Infect..

[45] Ding C., Patel D., Ma Y., Mann J.F.S., Wu J., Gao Y. (2021). Employing Broadly Neutralizing Antibodies as a Human Immunodeficiency Virus Prophylactic & Therapeutic Application. Front. Immunol..

[46] Hong J., Wang Q., Wu Q., Chen J., Wang X., Wang Y., Chen Y., Xia N. (2021). Rabbit Monoclonal Antibody Specifically Recognizing a Linear Epitope in the Rbd of Sars-Cov-2 Spike Protein. Vaccines.

[47] Ravichandran S., Coyle E.M., Klenow L., Tang J., Grubbs G., Liu S., Wang T., Golding H., Khurana S. (2020). Antibody Signature Induced by SARS-CoV-2 Spike Protein Immunogens in Rabbits. Sci. Transl. Med..

[48] Verkhivker G., Agajanian S., Oztas D., Gupta G. (2021). Dynamic Profiling of Binding and Allosteric Propensities of the SARS-CoV-2 Spike Protein with Different Classes of Antibodies: Mutational and Perturbation-Based Scanning Reveals the Allosteric Duality of Functionally Adaptable Hotspots. J. Chem. Theory Comput..

[49] Wrapp D., Wang N., Corbett K.S., Goldsmith J.A., Hsieh C.L., Abiona O., Graham B.S., McLellan J.S. (2020). Cryo-EM Structure of the 2019-NCoV Spike in the Prefusion Conformation. Science.

[50] Henderson R., Edwards R.J., Mansouri K., Janowska K., Stalls V., Gobeil S.M., Kopp M., Li D., Parks R., Hsu A.L. (2020). Controlling the SARS-CoV-2 spike glycoprotein conformation. Nat. Struct. Mol. Biol..

[51] Mannar D., Saville J.W., Sun Z., Zhu X., Marti M.M., Srivastava S.S., Berezuk A.M., Zhou S., Tuttle K.S., Sobolewski M.D. (2022). SARS-CoV-2 Variants of Concern: Spike Protein Mutational Analysis and Epitope for Broad Neutralization. Nat. Commun..

[52] Díaz-Salinas M.A., Li Q., Ejemel M., Yurkovetskiy L., Luban J., Shen K., Wang Y., Munro J.B. (2022). Conformational Dynamics and Allosteric Modulation of the SARS-CoV-2 Spike. eLife.

[53] Anasir M.I., Poh C.L. (2019). Structural Vaccinology for Viral Vaccine Design. Front. Microbiol..

[54] McCallum M., De Marco A., Lempp F.A., Tortorici M.A., Pinto D., Walls A.C., Beltramello M., Chen A., Liu Z., Zatta F. (2020). N-Terminal Domain Antigenic Mapping Reveals a Site of Vulnerability for SARS-CoV-2. Cell.

[55] Chi X., Yan R., Zhang J., Zhang G., Zhang Y., Hao M., Zhang Z., Fan P., Dong Y., Yang Y. (2020). A Neutralizing Human Antibody Binds to the N-Terminal Domain of the Spike Protein of SARS-CoV-2. Science.

[56] Chen Y., Lu S., Jia H., Deng Y., Zhou J., Huang B., Yu Y., Lan J., Wang W., Lou Y. (2017). A Novel Neutralizing Monoclonal Antibody Targeting the N-Terminal Domain of the MERS-CoV Spike Protein. Emerg. Microbes Infect..

[57] Bowen J.E., Park Y.-J., Stewart C., Brown J.T., Sharkey W.K., Walls A.C., Joshi A., Sprouse K.R., McCallum M., Tortorici M.A. (2022). SARS-CoV-2 Spike Conformation Determines Plasma Neutralizing Activity Elicited by a Wide Panel of Human Vaccines. Sci. Immunol..

[58] Williams J. (2013). Vector Design for Improved DNA Vaccine Efficacy, Safety and Production. Vaccines.

[59] Oliveira S.C., Rosìnha G.M.S., De-Brito C.F.A., Fonseca C.T., Afonso R.R., Costa M.C.M.S., Goes A.M., Rech E.L., Azevedo V. (1999). Immunological Properties of Gene Vaccines Delivered by Different Routes. Brazilian J. Med. Biol. Res..

[60] Bauer G. (2021). The Potential Significance of High Avidity Immunoglobulin G (IgG) for Protective Immunity towards SARS-CoV-2. Int. J. Infect. Dis..

[61] Struck F., Schreiner P., Staschik E., Wochinz-Richter K., Schulz S., Soutschek E., Motz M., Bauer G. (2021). Vaccination versus Infection with SARS-CoV-2: Establishment of a High Avidity IgG Response versus Incomplete Avidity Maturation. J. Med. Virol..

[62] Iwasaki A., Yang Y. (2020). The Potential Danger of Suboptimal Antibody Responses in COVID-19. Nat. Rev. Immunol..

[63] Puschnik A., Lau L., Cromwell E.A., Balmaseda A., Zompi S., Harris E. (2013). Correlation between Dengue-Specific Neutralizing Antibodies and Serum Avidity in Primary and Secondary Dengue Virus 3 Natural Infections in Humans. PLoS Negl. Trop. Dis..

[64] Gaspar E.B., De Gaspari E. (2021). Avidity Assay to Test Functionality of Anti-SARS-CoV-2 Antibodies. Vaccine.

[65] Zhou R., To K.K.-W., Wong Y.-C., Hung I.F.-N., Yuen K.-Y., Chen Z. (2020). Acute SARS-CoV-2 Infection Impairs Dendritic Cell and T Cell Responses. Immunity.

[66] Khatri I., Staal F.J.T., van Dongen J.J.M. (2020). Blocking of the High-Affinity Interaction-Synapse Between SARS-CoV-2 Spike and Human ACE2 Proteins Likely Requires Multiple High-Affinity Antibodies: An Immune Perspective. Front. Immunol..

[67] Correa V.A., Rodrigues T.S., Portilho A.I., Trzewikoswki de Lima G., De Gaspari E. (2021). Modified ELISA for Antibody Avidity Evaluation: The Need for Standardization. Biomed. J..

[68] Verma J., Subbarao N. (2020). Insilico Study on the Effect of SARS-CoV-2 RBD Hotspot Mutants’ Interaction with ACE2 to Understand the Binding Affinity and Stability. Virology.

[69] Tanaka S., Nelson G., Olson C.A., Buzko O., Higashide W., Shin A., Gonzalez M., Taft J., Patel R., Buta S. (2021). An ACE2 Triple Decoy That Neutralizes SARS-CoV-2 Shows Enhanced Affinity for Virus Variants. Sci. Rep..

[70] Kim S., Liu Y., Lei Z., Dicker J., Cao Y., Zhang X.F., Im W. (2021). Differential Interactions between Human ACE2 and Spike RBD of SARS-CoV-2 Variants of Concern. J. Chem. Theory Comput..

